# The lipid droplet: A conserved cellular organelle

**DOI:** 10.1007/s13238-017-0467-6

**Published:** 2017-09-14

**Authors:** Congyan Zhang, Pingsheng Liu

**Affiliations:** 1 National Laboratory of Biomacromolecules, CAS Center for Excellence in Biomacromolecules, Institute of Biophysics, Chinese Academy of Sciences, Beijing, 100101 China; 2 University of Chinese Academy of Sciences, Beijing, 100049 China

**Keywords:** lipid droplet, conserved organelle, lipid metabolism, nucleic acid handling

## Abstract

The lipid droplet (LD) is a unique multi-functional organelle that contains a neutral lipid core covered with a phospholipid monolayer membrane. The LDs have been found in almost all organisms from bacteria to humans with similar shape. Several conserved functions of LDs have been revealed by recent studies, including lipid metabolism and trafficking, as well as nucleic acid binding and protection. We summarized these findings and proposed a hypothesis that the LD is a conserved organelle.

## INTRODUCTION

The lipid droplet (LD) is a multi-functional organelle with unique structure that distinguishes it from other cellular organelles (Murphy and Vance, 1999; Martin and Parton, 2006; Farese and Walther, 2009; Welte, 2015). Since discovered in 1674 by Van Leeuwenhoek, the LD has been found to be an organelle necessary for many cellular functions that are essential for the organismic energy homeostasis, and more importantly for human health and aging. In addition to its role in lipid storage and metabolism (Cao et al., 2008; Cohen et al., 2011), recent studies have revealed that the LD is critical for membrane trafficking (Liu et al., 2004; Bartz et al., 2007), protein storage (Li et al., 2012) and degradation (Ploegh, 2007), and has a key role in hepatitis C virus (HCV) replication and assembly (Miyanari et al., 2007) and neurodegeneration (Liu et al., 2015). As important sites of neutral lipid storage and metabolism, the ectopic storage of lipids in LDs is a key cellular component in many diseases. On other hand, LDs in plants and oleaginous microorganisms provide not only food oil but also feedstock for biodiesel and industrial oil (Murphy, 2001; Alvarez and Steinbuchel, 2002; Murphy, 2012; Chen et al., 2014).

## LIPID DROPLETS EXIST FROM BACTERIA TO HUMANS

LDs are found in almost all organisms from bacteria to humans (Murphy, 2012; Waltermann et al., 2005). So far, except for knowing that LDs exist in all eukaryotic cells, it is also reported that some actinobacteria and cyanobacteria contain LDs, such as the genera *Micromonospora*, *Dietzia*, *Nocardia*, *Rhodococcus*, *Mycobacterium*, *Gordonia*, some streptomycetes (Murphy, 2001; Murphy, 2012), *Nostoc punctiforme* (Peramuna and Summers, 2014), *Synechococcus lividus* (Edwards et al., 1968), *Anabaena variabilis* (Wolk, 1973), and *Synechocystis* sp. PCC 6803 (Van de Meene et al., 2006). In addition, in comparison with other bacterial microcompartments including protein-based and lipid-bilayer membrane-based (Cornejo et al., 2014; Bobik et al., 2015), the LD is an unique organelle due to its particular structure and composition: neutral lipid core, phospholipid monolayer membrane, and peripheral proteins (Martin and Parton, 2006; Ding et al., 2012). This unique property is conserved from bacteria to humans.

## THE STRUCTURE AND COMPOSITION OF LIPID DROPLETS ARE CONSERVED

The core content of LDs in bacteria and eukaryotic cells is neutral lipid. Although some LDs contain retinyl ester (O’Mahony et al., 2015), polyhydroxyalkanoate or wax ester (Murphy, 2012), triacylglycerol (TAG) and cholesterol ester (CE) are the major neutral lipids of LDs in most cells (Waltermann and Steinbuchel, 2005; Barbosa and Siniossoglou, 2017). The neutral lipid core is coated by a phospholipid monolayer membrane in bacteria and eukaryotes (Martin and Parton, 2006; Farese and Walther, 2009; Waltermann and Steinbuchel, 2005), although the phospholipid composition may be different (Chitraju et al., 2012). In addition to the conserved lipid contents, the resident proteins of the organelle, including microorganism lipid droplet small (MLDS) and eukaryotic PERILIPIN (PLIN) family proteins (Kimmel et al., 2010), display conserved properties including the ability to target the phospholipid monolayer membrane and by the fact that they are all belong to apolipoprotein-like protein family (Yang et al., 2012).

These apolipoprotein-like proteins have also the ability to target LDs in diverse organisms, for example, mammalian LD proteins (PLINs) are targeted to LDs in yeast (Rowe et al., 2016) and bacteria (Hanisch et al., 2006). The LD resident proteins in *C*. *elegans*, DHS-3 and MDT-28/PLIN1 (Chughtai et al., 2015) behave similarly to target mammalian LDs (Na et al. 2015). In addition, a *Drosophila* LD resident protein, LSD1/PLIN1 localizes to LDs in *C*. *elegans* (Liu et al., 2014). The LD resident proteins, human adipose differentiation-related protein (ADRP)/PLIN2, *C*. *elegans* MDT-28, and bacterial MLDS are all able to bind to adiposomes that contain a TAG core with a phospholipid (DOPC) monolayer to mimic LDs *in vitro* (Wang et al., 2016). The ability of these proteins to target LDs of other organisms indicates that this fundamental process is highly conserved.

## THE LIPID DROPLET IS A FUNCTIONALLY CONSERVED ORGANELLE FROM BACTERIA TO HUMANS

**Figure 1:**
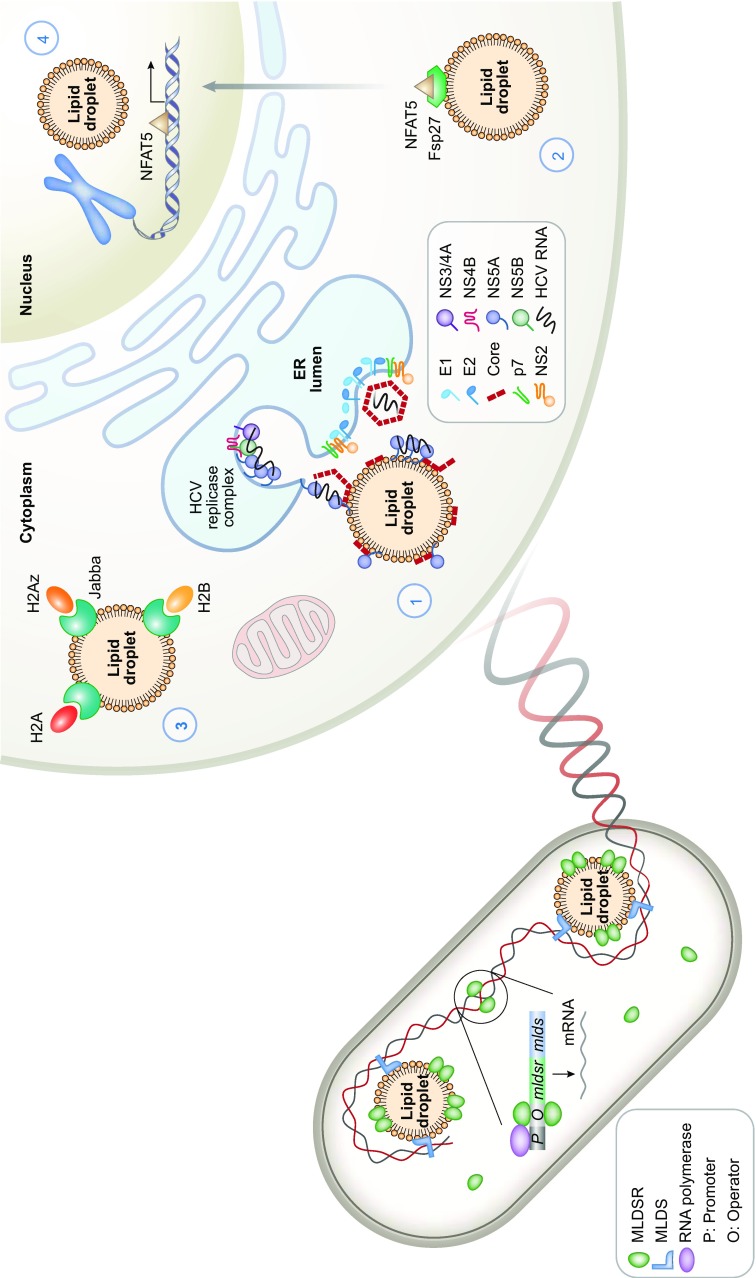
**The conserved lipid droplet functions of binding and regulating nucleic acids from bacterial to human cells**. In bacteria (left), LDs bind and protect genomic DNA via the major LD-associated protein, MLDS, which enhances the survival and adaptation of bacteria in extreme environments. Furthermore, a LD-associated transcriptional regulator, MLDSR, whose gene is in the same operon as *mlds*, induces or reduces the expression of MLDS when its cytosolic concentration is low or high, respectively. LDs have key role in transcriptional regulation by recruiting MLDSR to control its cytosolic concentration. Similar functions of LDs are also found in mammalian cells. In liver cells, hepatitis C virus is assembled around the LD surface and viral RNA is located to LDs through NS5A and core proteins. A hypothesis is proposed that after replication of viral RNA on the ER membrane, the newly synthesized RNA is moved by NS5A to the core protein on LDs, which triggers the initial viral assembly (right, part 1). In adipocytes, moreover, a transcriptional factor NFAT5 can be sequestered to LDs by Fsp27, which prevents its nuclear importation to initiate transcription (right, part 2). Several histones such as H2A, H2B, and H2Av are localized to LDs via the anchor protein Jabba in *Drosophila* (right, part 3). In addition, LDs are also present in the liver cell nucleus (right, part 4). The facts that both bacterial and mammalian LDs possess the function of nucleic acid handling indicate that LDs in living cells on earth are evolutionary conserved from prokaryotes to humans

Several functions of LDs are common through bacteria to humans, such as lipid storage and metabolism. However, the study of other functions of LDs, especially in bacteria, is insufficient. Recently, we found that the LDs in a bacterium, *Rhodococcus jostii* RHA1 (RHA1), bind to genomic DNA (Fig. 1) (Zhang et al., 2017) and protect it via their major protein, MLDS, which promotes bacterial survival under stress (Zhang et al., 2017). Furthermore, the study also reports that LDs are involved in transcriptional regulation via a LD-associated transcriptional regulator, MLDSR (Zhang et al., 2017). These two newly identified functions in bacteria suggest that LDs are unique endomembrane organelles involved in nucleic acid handling and facilitate bacterial survival in and adaptation to extreme environments (Zhang et al., 2017).

In eukaryotic and prokaryotic cells, LD proteomic analysis has revealed that RNA-binding proteins, ribosomal subunits, and/or translation factors are present on LDs (Ding et al., 2012; Sato et al., 2006; Zhang et al., 2012). Ribosomes and RNA are also found on mammalian LDs (Dvorak et al., 2003; Dvorak, 2005; Wan et al., 2007). In addition, HCV localizes and assembles around the LD surface (Fig. 1) (Miyanari et al., 2007; Shi et al., 2002; Gentzsch et al., 2013; Fiches et al., 2016). Furthermore, a mammalian homologue of the most abundant LD resident protein in *C*. *elegans*, MDT-28, is a mediator of RNA polymerase II (Zhang et al., 2012; Li et al., 2015). LDs in *Drosophila* store histones via the Jabba protein (Fig. 1) (Li et al., 2012, 2014; Cermelli et al., 2006). Interestingly, several recent studies identified LDs in the nuclei of mammalian cells (Fig. 1) (Layerenza et al. 1831; Wang et al., 2013; Ohsaki et al., 2016). LDs inhibit the translocation of NFAT5 to the nucleus via the LD-associated protein FSP27 and reduce NFAT5 transcriptional activity (Fig. 1) (Ueno et al., 2012). Altogether, these reports suggest that eukaryotic LDs partially mimic some nuclear functions, which is similar to bacterial LDs.

According to these previous studies, both bacterial and eukaryotic LDs are involved in nucleic acid handling, suggesting that the LD is a functionally conserved organelle. In the evolution from prokaryotes to eukaryotes, the most important feature is the protection of hereditary material (nuclear emergence). Thus, the function of bacterial LDs to protect and regulate nucleic acids indicates that they are analogous to the eukaryotic nuclear membrane.

Based on the extensive distribution, as well as the conservation of structure, composition, and functions of LDs from almost all living organisms, we propose a hypothesis that the LD is a conserved organelle from bacteria to humans (Fig. 1).
